# The Temptation of Zero Price: Event-Related Potentials Evidence of How Price Framing Influences the Purchase of Bundles

**DOI:** 10.3389/fnins.2018.00251

**Published:** 2018-04-20

**Authors:** Haiying Ma, Zan Mo, Huijun Zhang, Cuicui Wang, Huijian Fu

**Affiliations:** ^1^School of Management, Guangdong University of Technology, Guangzhou, China; ^2^Laboratory of Neuromanagement and Decision Neuroscience, Guangdong University of Technology, Guangzhou, China; ^3^School of Management, Hefei University of Technology, Hefei, China; ^4^Academy of Neuroeconomics and Neuromanagement, Ningbo University, Ningbo, China

**Keywords:** price framing, bundle, affect, purchase decision, ERPs, LPP

## Abstract

Studies have revealed that consumers are susceptible to price framing effect, a common cognitive bias, due to their limited capacity in processing information. The effect of price framing in a bundling context and its neural correlates, however, remain not clearly characterized. The present study applied the event-related potentials (ERPs) approach to investigate the role of price framing in information processing and purchase decision making in a bundling context. Three price frames were created with practically identical total prices (with a maximum difference of ¥0.1, which was about equal to 0.016 US dollars) for a bundle with two components, a focal product and a tie-in product. In normal price condition (NP), both the focal and tie-in products were offered at a normal discounted price; in zero price condition (ZP), the tie-in product was offered free while the total price of the bundle remained the same as NP; whereas in low price condition (LP), the tie-in product was offered at a low token price (¥0.1), and the focal product shared the same price as the focal product of ZP. The behavioral results showed a higher purchase rate and a shorter reaction time for ZP in contrast to NP. Neurophysiologically, enlarged LPP amplitude was elicited by ZP relative to NP, suggesting that ZP triggered a stronger positive affect that could motivate decision to buy. Thus, this study provides both behavioral and neural evidence for how different price framing information is processed and ultimately gives rise to price framing effect in purchase decision making.

## Introduction

Nowadays, with the proliferation of electronic commerce (e-commerce), consumers are exposed to all varieties of products with large amounts of information prior to making purchase decisions. Though perfect information may lead to a better decision, the limitation of human beings' ability to process information has made purchase decision a difficult task for consumers (Cheng et al., 2014). Human cognitive bias, which is likely to inflict negative effect upon decision quality, has thereby attracted substantial attention (Cheng et al., 2014; Gamliel et al., 2016).

The attribute framing effect is one of the most noted decision biases, which refers to the phenomenon that people show inconsistency in preferences or choices when identical attribute information is provided in different ways (Tversky and Kahneman, 1981). In marketing studies, price is a type of attribute information of a product and plays an import role in consumer decision making. A number of studies have probed into the influence of price framing on consumers' perceptions and purchase intentions (Chen et al., 1998; Khan and Dhar, 2010; Schmitz and Ziebarth, 2017). Chen et al. (1998) framed a discount in percentage terms (% off) vs. dollar terms ($ off) on differentially priced products, and suggested that a discount framed in dollar terms was more effective in enhancing consumer purchase intention of high-price product, whereas the opposite was true for the low-price product. Hamilton and Srivastava (2008) examined the pricing effect when the total price of a product and/or service was partitioned into two or more mandatory components. They found that consumers' reactions to price framing were moderated by the perceived consumption benefit of the components. Price framing effect was also observed in the bundling context (Khan and Dhar, 2010; Goh and Bockstedt, 2013). Bundling is a marketing practice of selling two or more products as a single package for a special price. It was noted that the purchase likelihood was higher for cross-category bundle when the price reduction was described as savings on the relatively hedonic item instead of as savings on the utilitarian item (Khan and Dhar, 2010). Moreover, consumers' intention to buy a customized bundle of information goods as well as the size of chosen bundling was greatly impacted by different multipart pricing schemes (Goh and Bockstedt, 2013).

Bundling has turned out to be a popular practice for both online and offline marketers and bundle pricing decision has become a major concern (Sheikhzadeh and Elahi, 2013; Shaddy and Fishbach, 2017). However, so far, the influence of bundle price framing upon consumer decision making has not been fully understood. It has been suggested that when people have to make a choice between two products, they tend to switch their preference from the preferred more expensive product to the less preferred but cheaper alternative when the latter is offered at zero price (namely zero-price effect), since a free product could give rise to positive affective reactions (Shampanier et al., 2007; Votinov et al., 2016; Hüttel et al., in press). In the multi-component bundling context, however, it's not clearly known how consumers would perceive and react if one price frame contains a free component while the other doesn't, provided that the total prices in different price frames are identical.

In addition, prior researches have generally adopted behavioral approaches to explore the price framing effect. Given the significant role of internal processes in driving cognitive bias, it is critical to gain insight into the associated underlying neural mechanisms, particularly how the price framing on bundles affects information processing in our brain and subsequent purchase decision-making. The application of neuroscientific approaches to marketing (i.e., neuromarketing) is promising in elucidating consumers' underlying thoughts, feelings, and intentions (Gajewski et al., 2016; Schaefer et al., 2016; Goodman et al., 2017; Hsu, 2017). Gajewski et al. (2016), for instance, investigated the electrophysiological brain activity during simulated purchase decisions of technical products offered at different price levels and observed enhanced conflict processing for counter-conformity decisions (buy an expensive product or not to buy a cheap one) vs. conformity decisions (buy a cheap product or not to buy an expensive one), which was reflected by longer reaction times, an increased N2 and a reduced P3. Besides, a few researchers have recently attempted to uncover the neurocognitive processes of attribute framing effect. Take Jin et al. (2017) as an example, they presented participants with two attribute frames regarding the contents of woolen products (i.e., positive frame was described as fabric contents in the products and negative frame described as artificial fabric contents in the products), and demonstrated that compared with negative frames, positive frames attracted less attention at the early stage (smaller P2 amplitude), evoked less cognitive conflict (smaller P2-N2 complex) and led to higher evaluation (larger LPP amplitude).

Therefore, the primary aim of the current study was to uncover the neural underpinnings of the price framing effect in bundle purchase decision-making by electrophysiological techniques. To attain this goal, two major price frames were created with the same total price for a bundle with two products, including a relatively expensive focal product and a relatively cheap tie-in product. In one price frame, both the focal and tie-in product were offered at a normal price (normal price condition, NP). In the second price frame, the tie-in product was offered at zero price while the total price of the bundle remained the same (zero price condition, ZP). Furthermore, a recent study reported an interesting finding that for price promotions offering product upgrades, it could be more effective when the upgrade was offered at a small token price (e.g., buy a Canon camera and upgrade its memory capacity from 16G to 32G for ¥0.1) rather than for free (Mao, 2016). We speculate that the tie-in product in a bundle might be treated as an “upgrade” in Mao's study. To test if Mao's findings could extend to a general bundling context, a third experimental condition was created such that the tie-in product was offered at a low token price (¥0.1, which was about equal to 0.016 US dollars at the time of experiment), whereas the focal product was offered at the same price as the focal product of ZP (low price condition, LP). Altogether, this study included three experimental condition (i.e., NP, ZP, and LP) with practically identical total prices (with a maximum difference of ¥0.1). During the experiment, participants were asked to view each bundle and determine if they would buy it or not while their scalp electroencephalogram (EEG) were recorded. According to prior literature on purchase decision making (Zhao et al., 2015; Goto et al., 2017), the late positive potential (LPP) is of particular interest to the current study.

The LPP is a positivity belonging to the P300 family, generally arises at about 400 ms after stimulus onset and lasts for several 100 ms (Schupp et al., 2000). The latencies of LPP vary across studies but tend to be predominant between 400 and 800 ms (Codispoti et al., 2012). LPP has a widespread scalp distribution from the frontal to the parietal sites with maxima over central-parietal sites. LPP is sensitive to motivationally relevant stimuli, and thought to reflect overt, post-perceptive deliberative processing related to stimulus significance (Olofsson et al., 2008). Emotionally significant stimuli (e.g., pleasant and unpleasant stimuli) has been found to trigger augmented LPP relative to neutral stimuli, suggesting enhanced activation of motivational system in the brain, increased resource allocation and sustained attentive processing for motivationally relevant stimuli (Schupp et al., 2004; Ferrari et al., 2011; Leite et al., 2012). Neuromarketing studies have revealed similar findings. Pozharliev et al. (2015), for instance, asked the participants to passively view pictures of luxury and basic branded products and noted increased LPP amplitude for luxury goods in the social context. Moreover, Goto et al. (2017) designed a virtual shopping task which revealed a positive relationship between LPP amplitude and subjective preferences of products. Consequently, LPP could reflect preferences based on more elaborative and conscious cognitive processes (Goto et al., 2017).

In the current study, three different price frames were created. Previous studies have demonstrated that options with no downside (no cost) could elicit more positive affect, which serves as an input for consumer decision making (Shampanier et al., 2007; Baumbach, 2016; Votinov et al., 2016). Thus, we hypothesize that the positive affect induced by a free component in a bundle could facilitate purchase decisions such that ZP will lead to higher purchase rate and enhanced LPP amplitude compared to NP and LP.

## Methods

### Participants

Thirty-three healthy right-handed undergraduates from Guangdong University of Technology participated in the study. All participants were native Chinese speakers with normal or corrected-to-normal visual acuity and without any history of neurological disorders or mental diseases. The experiment conformed to the Declaration of Helsinki and was approved by the Internal Review Board of the Laboratory of Neuromanagement and Decision Neuroscience, Guangdong University of Technology. Participants provided written informed consent prior to the experiment and were paid for their participation after the experiment. Data from four participants were excluded, three for excessive artifacts during EEG recording and one for noticing the experimental manipulation and the purpose of the study, resulting in 29 valid participants (15 females) ranging in age from 19 to 23 years (mean ± SD = 20 ± 2.1).

### Experimental stimuli

We used color digital pictures of 90 products selected from JD.COM, one of the largest online retailers in China. A variety of products were included, such as food, drink, electronics, personal hygiene products, stationery and others, all of which were familiar to our participants. Forty-five bundles were created, each of which comprised two products, a relatively expensive focal product and a relatively cheap tie-in product. The two products in each bundle were functionally complementary or related (e.g., a power bank and a USB cable, a pack of coffee, and a mug). Three price frames were devised for each bundle. Therefore, there were 45 trials in each frame condition and 135 trials altogether. For NP, the original prices for each component of the bundle were calculated as the mean of the prices in two different online shops. In order to encourage the participants to buy bundles during the experiment, offered prices for each component in NP were discounted from the means of the prices by ~20% (Knutson et al., 2007; Goto et al., 2017). For ZP, the tie-in product was offered at zero price while the total price of the bundle remains the same as NP. For LP, the tie-in product was offered at a low token price (¥0.1) while the focal product of LP had the same price as the focal product of ZP.

### Experimental procedure

**Figure 1:**
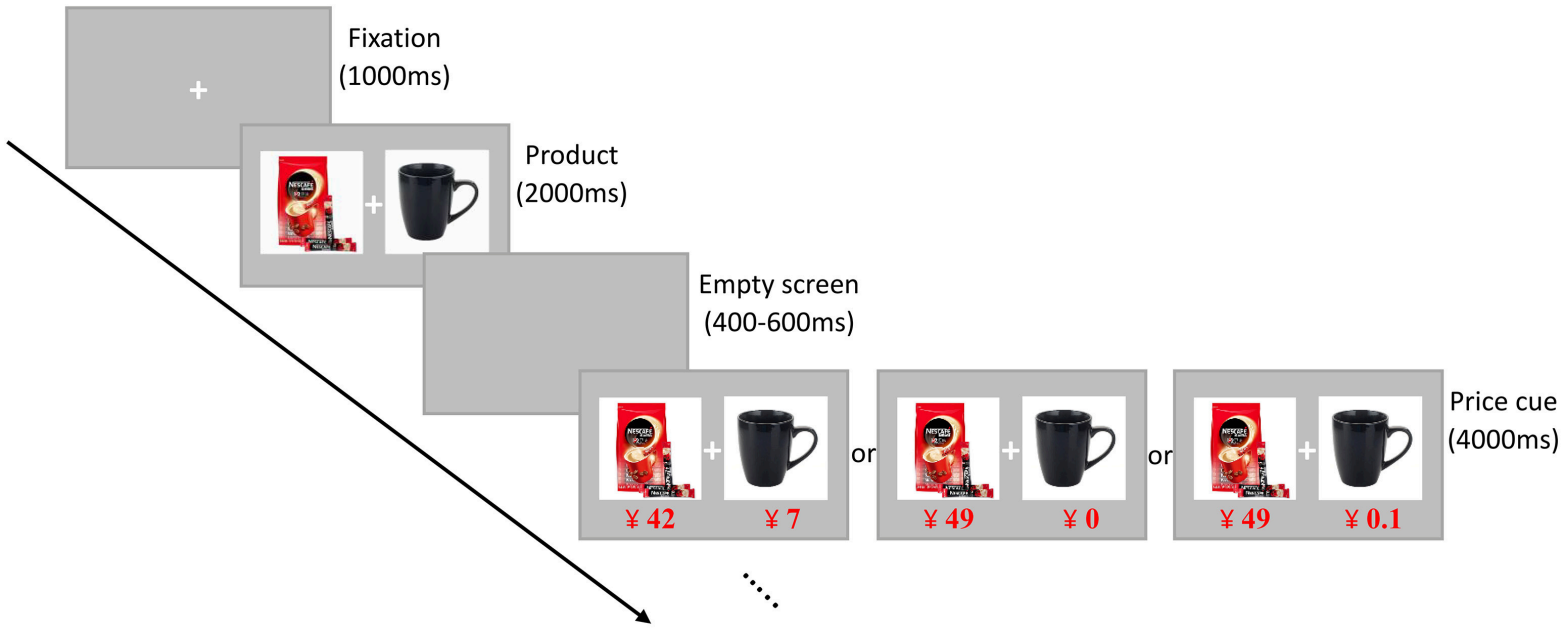
Schematic illustration of one trial of the experimental task. Participants were instructed to make a “buy” or “not buy” decision after the presentation of the bundle along with price information.

Participants were comfortably seated on a chair in a dimly lit, sound attenuated room. The stimuli were presented centrally on a 19-inch computer monitor (1,280 × 1,024 pixels, 60 HZ) against a gray background at a distance of 90 cm in front of the participants. E-Prime 2.0 software (Psychology Software Tools Inc., Pittsburgh, PA, USA) was used to deliver the stimuli and a keypad was provided for participants to make responses. Prior to the formal experiment, participants received instructions about the task and were tested for task comprehension in the practice trials. Participants got a virtual allocation of ¥70, which could be used to buy the bundles during the experiment. As illustrated in Figure 1, each trial began with a central fixation cross for 1,000 ms, which was followed by the presentation of a bundle for 2,000 ms with a visual angle of 8° × 3.7°. The focal product was placed to the left of the cross and the tie-in product the other side. Next, an empty screen was displayed for 400–600 ms randomly. Afterwards, the bundle was again presented with the prices displayed in red below each component for 4,000 ms, during which participants had to decide whether to buy the bundle or not at the offered prices. The response-to-hand assignments were counterbalanced across individuals such that half of them were instructed to press “1” for “buy” and “3” for “not buy” while the opposite was true for the other half. The virtual allocation was reset for every trial. The 135 trials were pseudorandomly assigned to three blocks, and the order of trials was pseudorandom within each block such that different price frames on an identical bundle did not appear within three consecutive trials. The experiment lasted for about 22 min. After finishing all trials, participants were asked if they were clearly aware of the experimental manipulation and the researchers' true intent. If a participant was aware of these, then the data from this participant would be excluded from further analysis.

To ensure the participants' motivational engagement in the shopping task, one trial was randomly selected to be implemented after the experiment (Knutson et al., 2007; Goto et al., 2017). If the participant chose to buy the bundle in that trial, then the bundle was later shipped to the participant, and cash “savings” corresponding to the initial allocation (¥70) minus the total price of the chosen bundle was paid to the participant. If not, the participant received the full allocation (¥70) as payment. This approach was used to maximize the realism of the shopping task because participants had a real chance of getting one of the “purchased” bundles, and cash saving was an inherent part of price-based shopping behavior.

Moreover, in order to minimize possible biases produced by strategies built upon buying only a small subset of products, and following previous research (Goto et al., 2017), participants were informed before the experiment that they would lose money on their final cash savings if they failed to buy a sufficient number of bundles. If the number of bundles bought was < 20, then ¥20 would be subtracted from the savings. If the number was between 20 and 24, ¥10 would be lost. If this number was between 25 and 29, ¥5 would be lost. If more than 30 bundles were bought, no money would be lost at the end. As a matter of fact, all participants bought more than 30 bundles and not any penalty was applied.

### EEG data recording and analysis

The EEG was recorded with eego amplifier, using a Waveguard EEG Cap with 64 Ag/AgCl electrodes mounted according to the extended international 10–20 system (both manufactured by ANT Neuro, Enschede, Netherlands). Channel data were online band-pass-filtered from 0.1 to 100 Hz and recorded at a sampling rate of 500 HZ. The left mastoid served as on-line reference, and the EEG was off-line re-referenced to the mathematically averaged mastoids. Impedances were kept below 10 kΩ throughout the experiment.

EEG data were pre-processed off-line using ASALab 4.10.1 software (ANT Neuro, Enschede, Netherlands). Ocular artifacts were identified and corrected with the eye movement correction algorithm used in the ASALab program. The EEG was digitally filtered with a low-pass filter at 30 Hz (24 dB/Octave) and segmented into epochs of 1,000 ms, time-locked to price onset and included a 200 ms pre-stimulus baseline. Trials containing amplifier clipping, bursts of electromyography activity, or peak-to-peak deflection exceeding ±100 V were excluded from averaging. ERP averages were created separately for each experimental condition (i.e., NP, ZP, and LP).

As expected, a pronounced LPP component was elicited by different price frames. According to the visual observation of the grand average waveforms as well as previous studies on purchase decision making (Goto et al., 2017), three electrodes (Cz, CPz, and Pz) distributed among the centro-parietal sites were selected for LPP analysis. The average amplitude of LPP in the time window of 400–600 ms after the onset of price stimulus was submitted to a 3 (price frame: NP, ZP, and LP) × 3 (electrode: Cz, CPz, and Pz) repeated-measure ANOVA. The Greenhouse-Geisser correction (Greenhouse and Geisser, 1959) was applied in case of violation of the sphericity assumption (uncorrected dfs and corrected *p*-values were reported), and the Bonferroni correction was used for multiple paired comparisons.

## Results

### Behavioral data

#### Purchase rate

**Figure 2:**
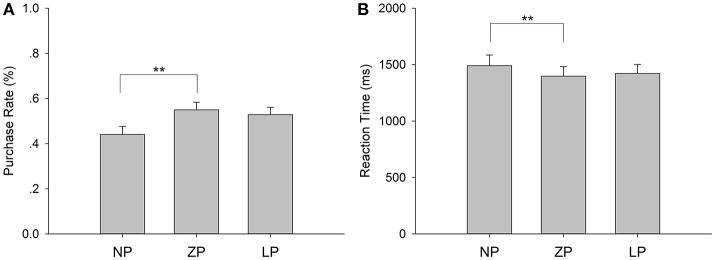
Behavioral results. **(A)** The purchase rate for each condition and **(B)** the RT for each condition. The error bars indicate standard error of the mean. ^**^p < 0.01.

Only trials that registered responses in <4 s after stimulus onset were included for behavioral analyses. The one-way repeated-measure ANOVA revealed a significant main effect of price frame on purchase rate, *F*_(2, 56)_ = 7.793, *p* = 0.007, $\eta_{\text{p}}^{2}$ = 0.218. As illustrated in Figure 2A, subsequent pairwise comparison indicated that participants made buy decisions more often in ZP (M = 0.550, S.E. = 0.033) compared to NP (M = 0.440, S.E. = 0.036, *p* = 0.009). But the contrast between ZP and LP (M = 0.529, S.E. = 0.032, *p* = 0.241), as well as the contrast between NP and LP (*p* = 0.065), was not significant.

#### Reaction time

The ANOVA showed a significant main effect of price frame on reaction time (RT), *F*_(2, 56)_ = 7.030, *p* = 0.004, $\eta_{\text{p}}^{2}$ = 0.201. As illustrated in Figure 2B, pairwise comparison indicated shorter RT for ZP (M = 1,387.390 ms, S.E. = 86.931) than NP (M = 1,484.370 ms, S.E. = 95.516, *p* = 0.004). However, the contrast between ZP and LP (M = 1,406.854 ms, S.E. = 79.764, *p* = 1.000), as well as that between NP and LP (*p* = 0.073), was not significant.

### ERP data

**Figure 3:**
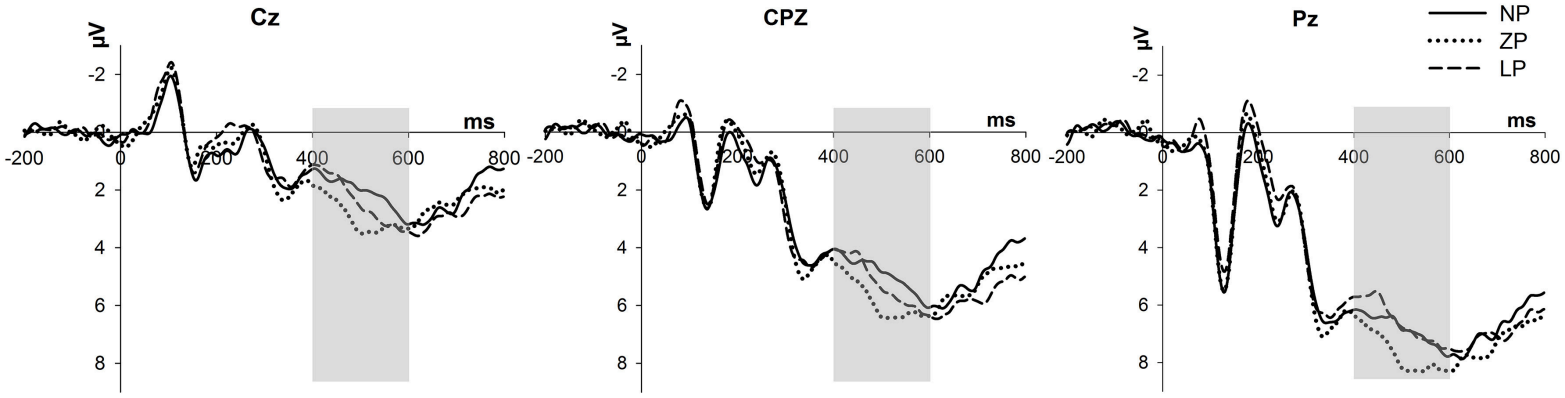
ERP results. Grand-averaged ERP waveforms from three central electrodes (Cz, CPZ, and Pz) time-locked to the onset of price stimulus. The gray rectangles denote the time window for LPP analysis. Positive voltage is plotted downwards.

As shown in Figure 3, the two-way repeated-measure ANOVA for LPP amplitude demonstrated a significant main effect of price frame, *F*_(2, 56)_ = 4.220, *p* = 0.020, $\eta_{\text{p}}^{2}$ = 0.131, and electrode, *F*_(2, 56)_ = 56.792, *p* = 0.000, $\eta_{\text{p}}^{2}$ = 0.670. The LPP amplitude elicited by ZP (M = 5.627 μV, S.E. = 0.696) was more positive than that by NP (M = 4.730 μV, S.E. = 0.725, *p* = 0.013). But similar to the behavioral results, there was neither statistically significant difference of LPP amplitudes between ZP and LP (M = 4.979 μV, S.E. = 0.687, *p* = 0.279), nor between NP and LP (*p* = 1.000). Furthermore, the LPP amplitudes at Cz (M = 2.687 μV, S.E. = 0.652), CPz (M = 5.448 μV, S.E. = 0.724), and Pz (M = 7.202 μV, S.E. = 0.783) differed from each other (*p*s < 0.05). However, the interaction between price frame and electrode was not significant, *F*_(4, 112)_ = 1.932, *p* = 0.110, $\eta_{\text{p}}^{2}$ = 0.065.

### Correlation analyses results

Two-tailed Pearson correlation analyses between the mean amplitude of LPP and RT were performed at the group level in order to explore if there were functional connections between one's brain activity and behavioral performance. It showed significant negative correlation between LPP amplitude and RT in NP (*r* = −0.381, p = 0.041), but no significant correlations between them in ZP (*r* = −0.102, *p* = 0.600), and LP (*r* = −0.320, *p* = 0.091).

## Discussion

The main goal of the present study was to elucidate how price framing influences purchase decision-making and its neural underpinnings. The behavioral results indicated that ZP led to a higher purchase rate and reduced RT than NP. Moreover, the ERP results showed larger LPP amplitude elicited by ZP in contrast to NP, providing neurophysiological evidence for the moderating effect of price framing on information processing and purchase decision making.

A remarkable price framing effect was discovered: people showed higher purchase rate when they were presented with bundles that contained a free component than when presented with bundles in which each component was offered at a normal price. Such a finding might be due to the positive affect induced by the zero-priced component (Shampanier et al., 2007; Nicolau, 2012; Nicolau and Sellers, 2012; Votinov et al., 2016). Previous studies have demonstrated that when people have to choose between two products, they tend to switch their preference from the preferred more expensive product to the less preferable but cheaper alternative when the cheaper option is offered for free (Shampanier et al., 2007; Votinov et al., 2016). A free offer could invoke a stronger positive affect and become extraordinary attractive since the zero price not only symbolizes no-cost but also implies extra benefit. This positive affect is used by consumers as a central input for decision making so that they're inclined toward the free option (Shampanier et al., 2007; Hüttel et al., in press). The zero price effect is not only confined to single products but also applies in multi-component contexts when one of the components becomes free (Nicolau and Sellers, 2012; Baumbach, 2016). In this study, stronger positive affect was evoked by the tie-in product when it was offered free rather than when offered at a normal price. This affect could extend to the evaluation of the bundle and made the bundle in ZP ostensibly more attractive. As a matter of fact, if consumers were rational persons, they would buy the same amount of bundles under different price frames since the total price of a bundle remained the same across different frames. We argue that people do not always act as rational economic models predict but instead they make decisions based substantially upon bounded rationality (Simon, 1956; Gigerenzer and Gaissmaier, 2011). For a purchase decision based on price information, affect may play a key role in the decision-making processes (Nicolau and Sellers, 2012; Somervuori and Ravaja, 2013), which give rise to the probability of non-rational economic behavior. When an individual's attention is focused on the positive aspect of a bundle (i.e., the zero-priced component), favorable associations could be evoked between the free component and its cost/benefit, leading to a higher purchase likelihood.

Moreover, people made purchase decisions faster in ZP rather than NP. It is proposed that RT is correlated with task difficulty and cognitive load (Wang et al., 2016). A shorter RT is generally suggestive of lower task difficulty and cognitive load (Cheng et al., 2014; Jin et al., 2017). In Jin et al. (2017)'s study, they asked participants to make purchase decisions in different attribute framing conditions (positive vs. negative), and found that the positive framing condition led to reduced RT relative to the negative framing condition, indicating that the stronger desirability of positive framing messages made purchase decisions easier. In the current study, the RT differentiation implicates that the task difficulty of ZP is lower than that of NP, and it entails less cognitive effort to make purchase decisions in ZP vs. NP. In line with Jin et al. (2017), ZP was more desirable to participants' expectation than NP, which might make purchase decision-making easier. A free component may lead people to feel more interested, elicit stronger positive affect, and accordingly capture a lot of attention. However, such an interpretation should be taken with caution since the lower difficulty of calculating a total price in ZP vs. NP could also contribute significantly to the shorter RT for ZP.

With regard to the ERPs component, we observed an effect of price framing on LPP in the 400–600 ms time window, with a topographical distribution across centro-parietal sites. LPP may be indicative of overt, post-perceptive deliberative cognitive processing related to stimulus significance (Olofsson et al., 2008). In consonance with the behavioral results, the neurophysiological results of this study showed larger LPP amplitude for ZP compared to NP, suggesting enhanced motivational engagement toward bundles with a free component, which increased resource allocation and facilitated sustained attentive processing (Schupp et al., 2004). A large number of studies have demonstrated that motivationally significant stimuli such as emotional stimuli, in contrast to neutral stimuli, lead to enlarged LPP amplitude (Schupp et al., 2004; Ferrari et al., 2011; Leite et al., 2012). In recent years, researchers have gained increasing interest in exploring the neural underpinnings of consumer emotion, attitude, and purchase intention (Pozharliev et al., 2015; Zhao et al., 2015; Bosshard et al., 2016; Goto et al., 2017; Wang et al., 2017). As Goto et al. (2017) noted, evaluating motivationally relevant consumer goods is quite similar to processing emotional stimuli in that they are usually associated with motivated attention. Zhao et al. (2015) reported that services with a high emotional value triggered a greater LPP amplitude, indicating that these services may motivate more positive emotions during purchase decision making. Pozharliev et al. (2015) examined the neural processes underlying passive viewing of luxury vs. basic branded goods, and showed increased LPP for luxury goods than for basic branded goods when the participants were together with another person, reflecting enhanced activation of motivational system in the brain for stimuli with higher emotional value. Furthermore, Goto et al. (2017) categorized ERP waveforms based on participants' preferences for a large variety of products and noted a positive relationship between LPP amplitude and subjective preferences, suggesting that subjective preferences were built on more elaborative and conscious cognitive processes. In a recent fMRI study, Votinov et al. (2016) engaged participants in a binary preference choice task with differentially priced products, which demonstrated a positive relationship between the activation of medial prefrontal cortex and the subjective happiness of obtaining free products and confirmed the role of affective evaluation in zero-price effect. As aforementioned in the current study, ZP might induce a stronger positive affect than NP because the former option contained a free component, which seemingly connoted no cost but extra value added to the bundle and made the offer highly attractive (Shampanier et al., 2007; Nicolau and Sellers, 2012; Votinov et al., 2016). Thereby, consistent with previous studies, the increased LPP amplitude for ZP vs. NP implies that ZP is motivationally more significant and is selected by the brain for heightened attentive processing, which to a large extent facilitates consumer purchase decision making, as evidenced by the higher purchase rate for ZP vs. NP.

It was worth noting that there were statistically significant differences at neither behavioral nor neural level between ZP and LP, as well as between NP and LP. The contrast between ZP and LP was of particular interest to this study. As Mao (2016) noted, in the context of price promotions offering product upgrades, it generated greater sales when the upgrades were offered at a low token price (e.g., buy a Canon camera and upgrade its memory capacity from 16G to 32G for ¥0.1) rather than for free (e.g., buy a Canon camera and upgrade its memory capacity from 16G to 32G for free). He suggested that when an upgrade was offered at a low price, its perceived attractiveness would be enhanced due to that the consumers tended to compare the token price with the upgrade's normative value and found the token price disproportionally small relative to the retail price; whereas when an upgrade was offered free, consumers were prone to evaluate it with the amount of required purchase. However, a token-priced upgrade would be no more favorable when consumers were asked to consider deal savings before evaluating the deal, which suppressed relative thinking (Mao, 2016). Thereby, we surmise that two reasons may account for the undifferentiated responses toward ZP and LP. Firstly, participants were exposed to different price frames in the current study, rendering it rather difficult to change their mindset rapidly, which implied that participants were inclined to resort to a sole criteria (e.g., perceived absolute savings) for decision making. Additionally, a number of products were used as stimuli in this study, which made it impossible for participants to estimate the normative value of the tie-in products (thought they were familiar with the products per se) and compare it with the token price within a limited time.

Based upon the above findings and discussion, this study also has practical implications for marketers and retailers. Bundling is a constant strategy in retailing in pursuit of not only more sales per order but also developing customer loyalty. The advance in e-commerce (including mobile e-commerce) has boosted the application of bundling strategy. Understanding the impact of the price of each component on consumer response to the bundle may prompt managers to make effective pricing decisions, especially in nowadays when e-commerce enables consumers to organize bundles by themselves. Given a fixed total price, setting a zero price for the tie-in product could evoke stronger positive affect than setting a normal discounted price for each component in the bundle, and lead the consumers more likely to make “buy” decisions. In other words, the free component in a bundle may act as a bait that draws attention from consumers and makes them more willing to give the bundle a try (Nicolau and Sellers, 2012).

However there are several limitations of our study which have to be acknowledged. First, we didn't measure positive/negative emotion directly via subjective ratings and the inference about the involvement of affective/emotional processes in price framing effect relied largely on observed electrophysiological activities during the task. This kind of reasoning is called reverse inference. Though reverse inference is extremely prevalent in cognitive neuroscience and neuromarketing, its validity has been regarded by some researchers as limited (e.g., Lee et al., 2017). Yet some researchers asserted that reverse inference was not intrinsically weak when applied with caution (e.g., Hutzler, 2014). Future studies are needed to replicate our findings by taking subjective measures of emotion into account, which allow a direct comparison between behavioral and neural results and draw conclusions in a more comprehensive way. Second, the difficulty of calculating the total price was not strictly controlled across different experimental conditions. It might be relatively easier to calculate the total price in ZP vs. NP, since the former condition contained a zero-priced component. Thus it could be argued that the differences in RT, LPP amplitude and purchase rate between ZP and NP might be partly due to the differentiated cognitive demand induced by calculating the total price. It was difficult to rule out the influence of task difficulty in the current research paradigm. However, we conjecture that the higher purchase rate in ZP vs. NP could not be simply attributed to the lower task difficulty since task difficulty has been found to be associated more often with cognitive and behavioral efficiency (as reflected in RT and accuracy) but less often with purchase decision outcome. In addition, contrary to the present study, higher task difficulty and cognitive load could also be accompanied by higher purchase rate (Wang et al., 2016).

## Conclusions

To summarize, the current study investigated the price framing effect and its associated underlying neural mechanisms in a bundling context, and demonstrated that different price frames were processed differently. The behavioral results showed that ZP, in contrast to NP, led to a higher purchase rate, suggesting a more positive affect elicited by the zero-priced component that motivated buying decision. Moreover, a shorter RT was observed for ZP instead of NP due to the lower processing difficulty. At the neural level, ZP triggered larger LPP amplitude than NP, which might be a result of the more positive affect induced by the former condition. Overall, this study took a preliminary step toward uncovering the neural correlates of price framing effect, which may benefit future marketing studies.

## Author contributions

HM and HF conceived and designed the study. HM collected and analyzed the data. HF, HM, and HZ interpreted the data and drafted the manuscript. HM, ZM, HZ, CW, and HF reviewed and edited the manuscript. HF administered the project.

### Conflict of interest statement

The authors declare that the research was conducted in the absence of any commercial or financial relationships that could be construed as a potential conflict of interest.
